# Immunological microenvironment differences between left and right colon cancer: dynamic interactions of CASC15+KLK6+ epithelial subpopulation with T cells and mast cells

**DOI:** 10.1097/JS9.0000000000003453

**Published:** 2025-09-09

**Authors:** Yibin Zhao, Hao Liu, Yu Wang, Jie Shen, Yang Le, Peiyuan Li, Yuxiang Zhao, Feng Gao, Wei Cui

**Affiliations:** a Department of Colorectal Surgery, Ningbo Medical Center Lihuili Hospital (Affiliated Lihuili Hospital of Ningbo University) Ningbo, China; b Institute of Bioengineering, Biotrans Technology Co., Ltd, Huzhou, China; c United New Drug Research and Development Center, Biotrans Technology Co., Ltd, Changsha, China; d Department of General Surgery (Colorectal Surgery), The Sixth Affiliated Hospital, Sun Yat-sen University, Guangzhou, China

**Keywords:** colorectal cancer, immune evasion, immune microenvironment, mast cell, single-cell RNA sequencing, T-cell exhaustion

## Abstract

**Background:**

Right-sided colon cancer (RCC) and left-sided colon cancer (LCC) exhibit distinct clinical behaviors and molecular characteristics, but immune microenvironmental differences remain unclear.

**Methods:**

Single-cell RNA sequencing was performed on three LCC and three RCC tumors. Validation studies included immunofluorescence on 70 patients (30 LCC and 40 RCC). Analytical methods comprised cellular heterogeneity assessment (Seurat, inferCNV), pseudotime trajectory reconstruction (Monocle2), pathway enrichment (GSVA), and cell-cell communication inference (CellChat).

**Results:**

RCC displayed significantly increased exhausted CD8^+^ T cells and decreased activated CD8^+^ T cells compared to the LCC. Concurrently, RCC showed reduced proportions of TNF^+^ mast cells (MCs) and increased VEGFA^+^ MCs. We identified a CASC15^+^KLK6^+^ epithelial subpopulation specifically enriched in RCC tumors, exhibiting high copy number variation scores, elevated malignant pathway activity, and a significant negative correlation with patient survival (r = − 0.2919, p = 0.0142). Enhanced intercellular communication involving MIF signaling (CD74/CXCR4/CD44) and CD99 pathways was observed between epithelial cells, T cells, and MCs in RCC.

**Conclusion:**

RCC harbors an immunosuppressive niche driven by T-cell exhaustion, MC reprogramming, and expansion of a malignant CASC15^+^KLK6^+^ epithelial subpopulation. Targeting MIF and CD99 signaling networks represents a potential therapeutic strategy for RCC.

**HIGHLIGHTS**

- Distinct immunosuppressive landscape in right-sided colon cancer (RCC): Elevated T cell exhaustion and depleted TNF^+^ mast cells (MCs) vs. left-sided tumors (LCC).
- CASC15^+^KLK6^+^ epithelial subpopulation defines RCC aggressiveness: Specific expansion correlating with poor survival, high CNV burden, and malignant pathway activation.
- MIF/CD99-mediated intercellular crosstalk: Dominant epithelial-T cell-MC communication driving immune evasion in RCC.

## Introduction

Colorectal cancer (CRC) is among the leading causes of cancer incidence and mortality worldwide, characterized by significant heterogeneity and a complex tumor microenvironment (TME)^[1,2]^. Despite the success of immunotherapeutic strategies such as immune checkpoint blockade (ICB) in a subset of CRC patients with deficient mismatch repair (dMMR) or high microsatellite instability (MSI-H), the majority of CRC patients remain unresponsive to such treatments. This highlights the presence of unique immunosuppressive features and tolerance mechanisms within the CRC immune microenvironment^[3,4]^. To advance precision medicine for CRC, a comprehensive understanding of its TME is of paramount importance.

The biological and clinical behaviors of CRC are significantly influenced by the anatomical location of the primary tumor. Right-sided colon cancer (RCC, originating from the cecum, ascending colon, and hepatic flexure) and left-sided colon cancer (LCC, originating from the splenic flexure, descending colon, and sigmoid colon) exhibit marked differences in genetic backgrounds, molecular characteristics, and therapeutic responses^[5–9]^. Notably, RCC is associated with poorer survival outcomes compared to LCC^[7,10–12]^. While prior studies have elucidated certain immune cell features in CRC^[8,13,14]^, there remains a critical gap in understanding the immune microenvironmental differences between RCC and LCC and their implications for tumor progression.

Recent advances in single-cell sequencing technology have provided powerful tools for dissecting cellular heterogeneity and interactions within complex TMEs. In CRC research, single-cell transcriptomics enables the identification of molecular characteristics and functional states of diverse immune and epithelial cell populations within tumors^[14–16]^. For example, it facilitates the analysis of immune cell subpopulations and their spatial distributions, shedding light on their interactions with tumor cells and mechanisms of immune evasion. However, systematic comparisons of subpopulation-specific characteristics and intercellular communication networks in the TMEs of the LCC and RCC remain limited.

The critical role of the immune microenvironment in tumor immune evasion and therapy has been widely studied. Research has shown that T cells dominate the immune microenvironment in both LCC and RCC^[14,15]^. Beyond adaptive immunity, innate immune pathways like TLR signaling contribute to CRC heterogeneity. Right-sided colon’s unique microbiome may activate TLR4 to promote pro-tumorigenic inflammation[17], potentially synergizing with T-cell dysfunction. Mast cells (MCs) also play multiple roles in the TME, as they can release various cytokines and chemical mediators,^[18–21]^ and regulate T cell activation and function. Studies have reported that the interaction between T cells and MCs can promote immune suppression or lead to the dysfunction of regulatory T cells and loss of self-immunity^[22,23]^. The complex interaction between T cells and MCs plays a crucial role in tumor immune regulation and progression. Investigating the dynamic roles of these immune cell subpopulations is of significant value in uncovering the immune evasion mechanisms in CRC, especially in RCC.

Building upon this background, the present study employs single-cell transcriptomics to comprehensively compare the immune microenvironment and cellular heterogeneity between LCC and RCC. Special focus was placed on the characteristics and interactions of tumor cells, T cell subpopulations, and MC subpopulations. The aim was to provide new insights into the potential immune evasion characteristics of RCC and offer theoretical support for future immunotherapy strategies.

The work has been reported in line with the TITAN (Transparency In The reporting of Artificial INtelligence) criteria[24]. No artificial intelligence tools were used in the conduct of the research or the development of this manuscript.

## Materials and Methods

### Human tumor samples

All patients had not received chemotherapy, radiotherapy, or targeted therapy prior to surgery. A total of six patients provided six samples. The clinical and pathological information is summarized in Supplemental Digital Content Table 1, available at, http://links.lww.com/JS9/F61.

### Integration and analysis of single-cell RNA sequencing data

Single-cell RNA sequencing (scRNA-seq) was employed to analyze CRC samples. Tissues from LCC (3 samples) and RCC (3 samples) were collected and dissociated into single cells. The 10x library was generated according to the manufacturer’s protocol of 10x genomics Single Cell 3’ Reagent Kits v2 (10x Genomics ChromiumTM). Data integration and preprocessing were performed using the R package Seurat (v4.1.0). Quality control (QC) standards included filtering cells with nFeature ≥ 100, nCounts ≥ 100, mitochondrial gene content < 20%, and nFeature < 3000 to ensure analytical accuracy. Post-normalization, genes with high intercellular variability were identified and visualized using feature variance plots. Principal component analysis (PCA) was performed to extract features from samples and genes. Using the top 30 principal components, neighboring cells were identified with the FindNeighbors function, clustered with FindClusters (resolution parameter: 0.6), and visualized using Uniform Manifold Approximation and Projection (UMAP) dimensionality reduction. Cell annotations were performed with established markers from previous study[25] and the Cellmarker2.0 database, followed by analyses of cell-type proportions. For single-cell level comparisons of gene expression, we used the FindMarkers function in Seurat with a threshold of |log2 fold change| > 0.25 and adjusted p-value < 0.05 (Benjamini-Hochberg correction).

### Analysis of T cell subpopulations

Annotated T cell populations were extracted, and PCA and UMAP analyses were reapplied to identify T cell heterogeneity. Subpopulations were annotated based on characteristic marker genes with references to cellmarker2.0 and literature. The proportions of T cell subpopulations in LCC and RCC were statistically compared to reveal potential functional differences across CRC types.

### Analysis of MC subpopulations

MC populations were isolated, and their heterogeneity was analyzed based on marker gene expression profiles. Tumor-associated MC markers, such as TNF and VEGFA[18], were used for functional annotation. The proportions of TNF^+^ and VEGFA^+^ MCs in LCC and RCC were compared to explore their functional roles in different cancer subtypes.

### Heterogeneity of epithelial cells

Epithelial cell data were extracted and annotated into subpopulations using known markers. InferCNV was used to assess copy number variation (CNV) characteristics of epithelial subpopulations, aiding in the identification of malignant epithelial cells and their genomic instability.

### Pseudotime trajectory analysis

Pseudotime trajectory analysis was conducted on epithelial subpopulations using Monocle2, reconstructing potential trajectories of malignant transformation. Branch-specific gene expression analysis was performed with BEAM to investigate dynamic changes in key genes during epithelial deterioration.

### HALLMARK pathway enrichment analysis

The GSVA package was used to calculate HALLMARK pathway scores for epithelial cell subpopulations, evaluating malignancy potential. Particular attention was paid to the CASC15^+^KLK6^+^EPC subpopulation and its unique malignant characteristics in RCC.

### Immunofluorescence detection of EPC subpopulation in LCC and RCC tissues

Immunofluorescence was performed on 30 LCC and 40 RCC tumor tissue samples to detect EPCAM and KLK6 double-positive cells. The density of double-positive cells (number of double-positive cells per unit tissue area) was analyzed for correlation with patient survival (in months). Statistical analyses and visualization were performed with GraphPad Prism 9, and p < 0.05 was considered statistically significant.

### Cell-cell communication analysis

Cell-cell communication analysis was performed using CellChat (v1.6.1) with default parameters, which infers biologically significant interactions by integrating ligand-receptor interactions with prior knowledge of signaling pathways. The communication probability was calculated based on the expression of ligand-receptor pairs in the CellPhoneDB database (v4.0) and their interaction strength. The aggregated communication network was then calculated by summarizing the communication probability of all ligand-receptor pairs associated with each signaling pathway. The “communication intensity” for a given pathway was defined as the sum of probabilities across all ligand-receptor pairs divided by the number of cell pairs. Statistical significance was assessed by permutation testing (1000 iterations) to compare observed communication strengths with random distributions. Probabilities and intensities of signaling pathways and ligand-receptor interactions were compared between LCC and RCC. Differences in the number and strength of communication signals were visualized using bubble plots, heatmaps, and other visualization tools.

## Results

### scRNA-seq data integration and cellular atlas

**Figure 1.**
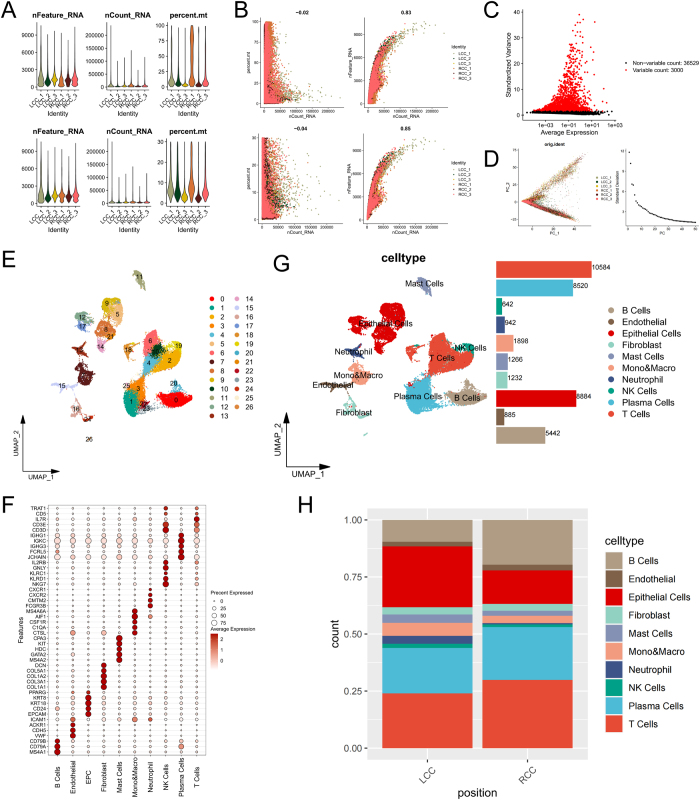
scRNA-seq data integration and cellular atlas. (A) UMI counts, gene numbers, and mitochondrial gene percentages before (top) and after (bottom) filtering. (B) Scatterplots showing mitochondrial gene percentages versus UMI counts (left) and gene numbers versus UMI counts (right) pre- and post-quality control. (C) Identification of 3000 highly variable genes. (D) PCA dimensionality reduction (based on 3000 highly variable genes). (E) UMAP plot displaying 20 cell clusters in low-dimensional space. (F) Bubble plot of marker gene expression for cell annotation. Cell type annotation using canonical markers (10 major types), and the specific number of cells was indicated. (H) Stacked bar plot of cell-type proportions in LCC and RCC samples (n = 3/group, mean proportions).

Our integrated scRNA-seq analysis of three LCC and three RCC tumors captured 53 752 cells (post-QC: 40 295 cells) and 39 529 genes. Figure 1A illustrates changes in the number of genes per cell, UMI counts, and mitochondrial gene percentages before and after filtering. A scatterplot of mitochondrial gene percentages versus UMI counts (Fig. 1B) highlights the improved data quality post-filtering. Using highly variable gene identification, 3000 highly variable genes were identified (Fig. 1C). PCA identified 30 principal components capturing >85% cumulative variance, with an inflection point at PC30 in the elbow plot (Fig. 1D). These PCs were selected for downstream analysis as they encompassed major biological variation while minimizing technical noise. UMAP dimensionality reduction delineated 20 cell clusters (Fig. 1E). Through cell annotation, 10 cell types were identified, including B cells, endothelial cells, epithelial cells, fibroblasts, MCs, monocytes/macrophages, neutrophils, NK cells, plasma cells, and T cells. The annotated UMAP plot is shown in Figure 1F, while the cell-type composition is summarized in the stacked bar chart in Figure 1G. The results indicate that T cells constitute the most abundant immune cell population in both LCC and RCC, followed by B cells, while MCs are the least abundant.

**Figure 2.**
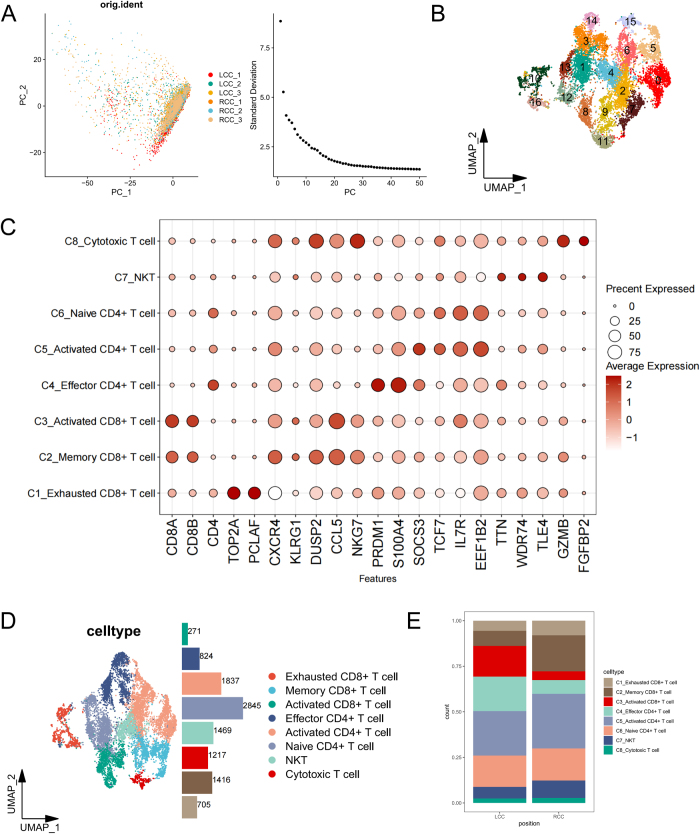
Heterogeneity analysis of T cell subsets in LCC and RCC. (A) PCA dimensionality reduction of T cells (Seurat-normalized data). (B) UMAP clustering of T cell subsets. (C) Bubble plot for T cell subset annotation (expression level: dot size; significance: color). (D) UMAP distribution of annotated T cell subsets, and the specific number of cells was indicated. (E) Stacked bar chart showing proportional differences of T cell subsets between LCC and RCC (n = 3/group, mean proportions).

### Heterogeneity analysis of T cell subsets in LCC and RCC

Given that T cells were the dominant population in both LCC and RCC immune microenvironments, we further investigated the differences in T cell subsets within the immune microenvironment of LCC and RCC. We extracted T cell data and performed further PCA (Fig. 2A) as well as subset analysis (Fig. 2B). Based on T cell marker genes from the CellMarker2.0 database (Fig. 2C), multiple T cell subsets were identified, including activated CD4^+^ T cells, activated CD8^+^ T cells, cytotoxic T cells, effector CD4^+^ T cells, exhausted CD8^+^ T cells, memory CD8^+^ T cells, naive CD4^+^ T cells, and natural killer T (NKT) cells. The UMAP distribution of these subsets is shown in Figure 2D. Proportion analysis revealed that RCC exhibited significantly higher levels of exhausted and memory CD8^+^ T cells, along with reduced proportions of activated CD8^+^ T cells and effector CD4^+^ T cells compared to LCC (Fig. 2E). These differences may indicate the presence of stronger immunosuppression and immunodepletion signatures in the immune microenvironment of RCC, providing new insights into immune escape mechanisms in CRC.

### Characterization of MC subpopulations in LCC and RCC

**Figure 3.**
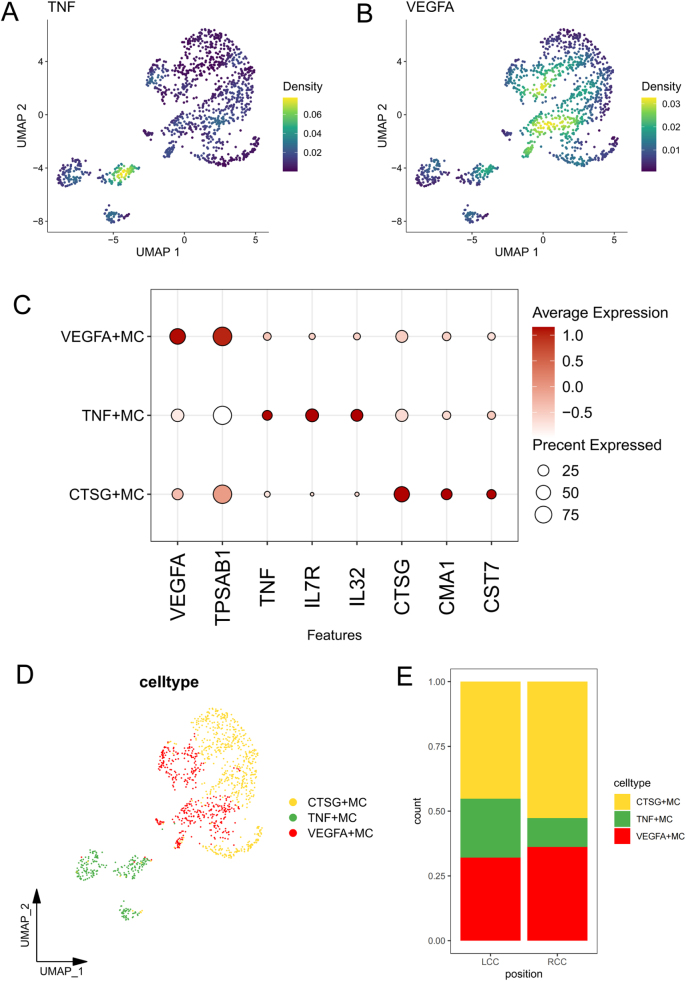
Characterization of mast cell subpopulations. (A) Expression distribution of TNF in mast cell subpopulations. (B) Expression distribution of VEGFA in mast cell subpopulations. (C) Marker gene expression bubble plot for mast cell subpopulations. (D) UMAP annotation of mast cell subpopulations (TNF^+^MC, VEGFA^+^MC, CTSG^+^MC). (E) Proportions of mast cell subpopulations in RCC and LCC.

Macrophages and dendritic cells have been well-studied in CRC,^[26–28]^ while MCs emerging as key regulators of angiogenesis and immune suppression[23]. Our focus on MCs stems from their understudied role in RCC-specific microenvironment remodeling. MCs play a multifaceted role in TME, regulating T cell activation and immune responses. After exploring the immune characteristics of T cell subsets, we further analyzed the heterogeneity of MC subsets in the immune microenvironment of LCC and RCC. According to a previous pan-cancer single-cell transcriptomic atlas of tumor-infiltrating myeloid cells, the proportion of TNF^+^ MCs and VEGFA^+^ MCs was significantly associated with cancer prognosis[18]. TNF^+^ MCs recruit immune cells through pro-inflammatory actions, while VEGFA^+^ MCs support tumor growth and suppress immune responses. A higher TNF^+^/VEGFA^+^ ratio correlates with better prognosis. In our dataset, we analyzed the expression distributions of TNF and VEGFA across MC subpopulations (Fig. 3A, B), further annotating high-expression TNF or VEGFA MC subpopulations as TNF^+^MC or VEGFA^+^MC, respectively. VEGFA^+^ cells also highly express TPSAB1, while TNF^+^ cells express IL7R and IL32, highlighting the functional differences of these subpopulations in regulating the microenvironment. Other subpopulations express markers such as CTSG, CMA1, and CST7, identifying them as CTSG^+^ cells (Fig. 3C). Figure 3D displays the UMAP plot showing the distribution of these three MC subpopulations. Further analysis of the MC subpopulation ratios revealed that compared to LCC, the proportion of TNF^+^ MCs was significantly reduced in RCC, while VEGFA^+^ MCs were recruited, leading to a dramatic decrease in the TNF^+^/VEGFA^+^ cell ratio in RCC patients (Fig. 3E). This change may drive enhanced angiogenesis and exacerbated immune evasion in RCC, further reinforcing its immunosuppressive characteristics and closely correlating with the poor prognosis observed in these patients.

### Identification of malignant epithelial subpopulations

**Figure 4.**
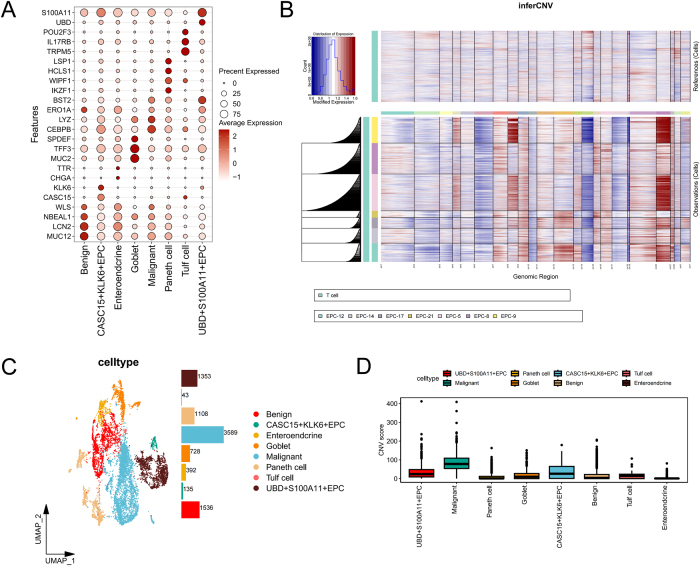
Malignant epithelial cell subpopulation identification. (A) Bubble plot of marker gene expression for epithelial cell subpopulations. (B) Copy number variation (CNV) analysis of epithelial cells (inferCNV scores). (C) UMAP annotation of epithelial cell subpopulations, distinguished by color (eight subsets), and the specific number of cells was indicated. (D) CNV score comparison across epithelial cell subpopulations, identifying malignant epithelial subpopulations.

he heterogeneity of tumor epithelial cells (EPCs) plays a crucial role in tumor initiation, progression, and immune evasion. The deterioration of epithelial cells is closely associated with tumor progression, but the signaling pathway differences in epithelial dedifferentiation between LCC and RCC remain unclear. The differential signals between these subgroups, which may contribute to the significant survival differences between LCC and RCC patients, require further elucidation. To further explore epithelial cell heterogeneity in LCC and RCC, we conducted subpopulation analysis. Based on previous literature[25] and markers from the CellMarker2.0 database (Fig. 4A), along with inferCNV scores (Fig. 4B), we identified eight subpopulations of epithelial cells, including Benign, CASC15^+^KLK6^+^EPC, Enteroendocrine, Goblet, Malignant, Paneth cell, Tulf cell, and UBD^+^S100A11^+^EPC (Fig. 4C). CNV score analysis (Fig. 4D) revealed that Malignant, CASC15^+^KLK6^+^EPC, and UBD^+^S100A11^+^EPC exhibited significantly higher CNV scores than other subpopulations, indicating that these cells have a higher malignant potential and may play a key role in tumor progression.

### Dynamic gene changes in epithelial cell dedifferentiation

**Figure 5.**
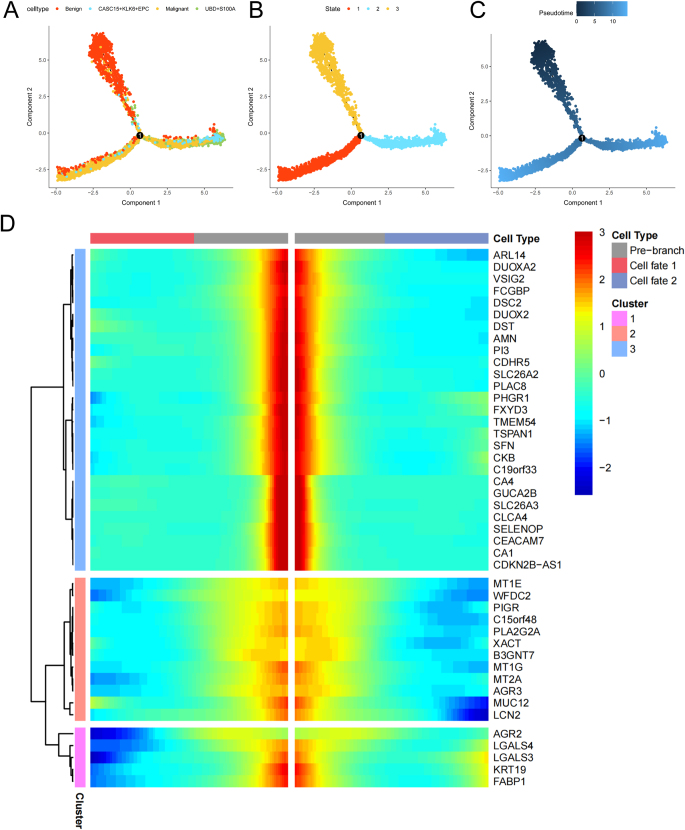
Pseudotemporal trajectory of cell dedifferentiation. (A) Pseudotime trajectory of epithelial subpopulations (root: benign cells). (B) Cell state distribution along pseudotime. (C) Branching structure of pseudotime trajectory (Node 1 bifurcation to malignant/CASC15^+^KLK6^+^EPC). (D) Heatmap of branch-differentiated gene expression changes over pseudotime.

To analyze the dynamic gene changes during epithelial cell dedifferentiation, we extracted various cell populations, including Benign epithelial cells, CASC15^+^KLK6^+^EPC, UBD^+^S100A11^+^EPC, and Malignant epithelial cells, and performed pseudotime analysis using Monocle2. This allowed us to infer the dedifferentiation pattern of epithelial cells. The results shown in Figure 5A to C present the distribution of different subpopulations and states of EPC in two-dimensional space, along with differentiation trajectories along pseudotime. Using Benign epithelial cells as the differentiation starting point, we observed that the cell population began to extend in different differentiation directions at node 1. Specifically, one direction was dominated by Malignant cells, while the other mainly comprised CASC15^+^KLK6^+^EPC and UBD^+^S100A11^+^EPC, which are both malignant epithelial cells. To identify key genes driving differentiation from Benign epithelial cells to Malignant, CASC15^+^KLK6^+^EPC, or UBD^+^S100A11^+^EPC, we performed BEAM analysis to filter genes associated with branching. As shown in Figure 5D, the heatmap illustrates the expression of these key genes over pseudotime. Many genes involved in maintaining cellular homeostasis were significantly downregulated during differentiation (whether toward the left or right). For example, the GUCA2B gene, which encodes a protein involved in hydrolysis to generate functional proteins, may regulate salt and water homeostasis in the intestines and kidneys; the TSPAN1 gene, which encodes a transmembrane protein, is involved in cellular development, activation, growth, and signaling. The downregulation of these genes suggests that during epithelial cell dedifferentiation, the maintenance of cellular homeostasis is gradually disrupted, setting the stage for malignant transformation.

### Pathway activity in malignant epithelial subpopulations

**Figure 6.**
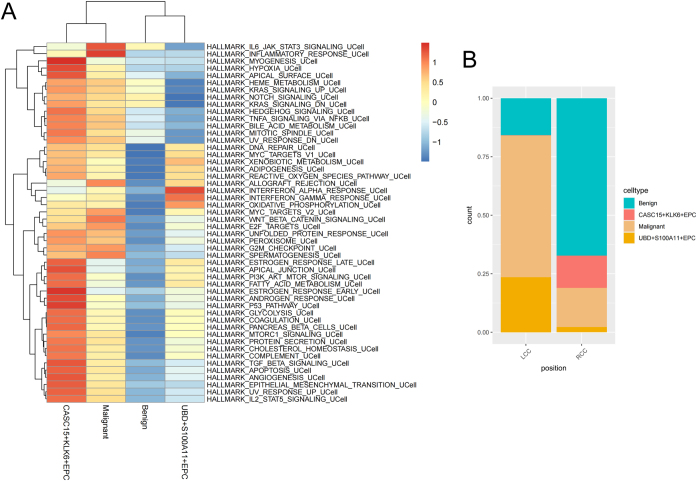
HALLMARK pathway analysis of EPC subpopulations. HALLMARK pathway enrichment heatmap (GSVA scores, row-scaled). (B) Proportions of EPC subpopulations in LCC and RCC.

In further exploring the biological characteristics of EPCs, we conducted HALLMARK pathway enrichment analysis (Fig. 6A) and found that CASC15^+^KLK6^+^EPC scored significantly higher than other subpopulations across most tumor progression-related pathways, suggesting that this subpopulation has higher malignant potential. The stacked cell proportion plot in Figure 6B further shows the specific enrichment of CASC15^+^KLK6^+^EPC in RCC, and its high malignant characteristics may be consistent with the poor clinical prognosis of RCC, suggesting that it may play an important role in RCC progression.

### Specific localization and expression of CASC15^+^KLK6^+^EPC in RCC

**Figure 7.**
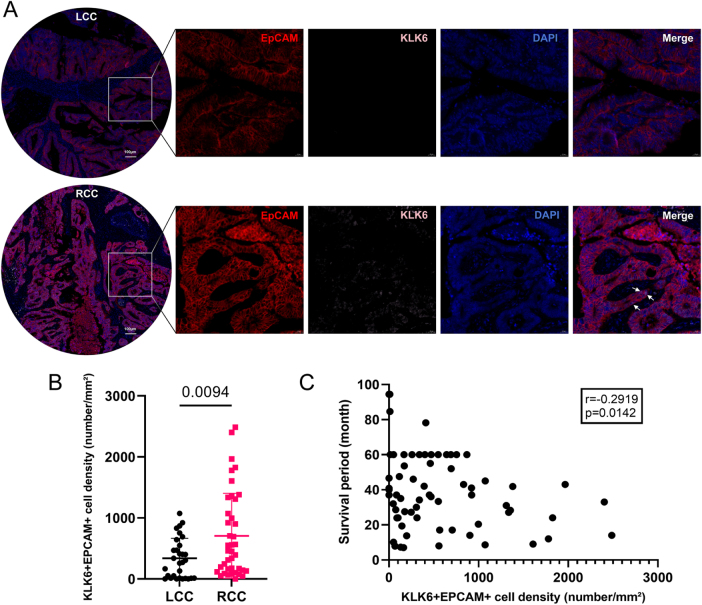
Clinical validation of CASC15^+^KLK6^+^EPC. Immunofluorescence staining in RCC and LCC tissues (EPCAM^+^KLK6^+^ double-positive cells; scale bar: 100 μm). Density quantification of double-positive cells (RCC vs. LCC: p = 0.0094, n =40 vs. 30). Negative correlation between double-positive cell density and patient survival (r = − 0.29, p = 0.014; Pearson correlation).

Given the exploratory nature of this scRNA-seq study with a limited sample size (n =3 per group), we focused our analysis on identifying potential differences in the TME between LCC and RCC. Key findings were validated in a larger cohort using immunofluorescence (n = 30/40). The CASC15^+^KLK6^+^EPC subpopulation highly expresses CASC15 and KLK6. CASC15 is a long non-coding RNA (lncRNA) that is highly expressed in various cancers, particularly in melanoma, neuroblastoma, and gastrointestinal cancers. CASC15 promotes tumor cell proliferation, migration, and invasion by regulating the expression of multiple oncogenes and tumor suppressor genes. KLK6 encodes a serine protease, part of the kallikrein family, which is also highly expressed in several cancers, including breast, ovarian, and CRCs, promoting tumor cell invasion and migration. The high co-expression of both suggests that this cell subpopulation has greater invasive and metastatic potential. Their CNV features and high scores in HALLMARK pathways further support this hypothesis. To validate this, we performed double immunofluorescence staining to assess the expression and localization of KLK6^+^EPC in 30 LCC and 40 RCC tumor tissue samples (Fig. 7A). The results showed that compared to LCC, the density of EPCAM^+^KLK6^+^ double-positive cells was significantly higher in RCC tumor tissues (Fig. 7B). Moreover, correlation analysis with survival period (months) revealed that the density of double-positive cells was significantly associated with patient survival (r = − 0.2919, p = 0.0142, Figure 7C). These findings support the high expression of this EPC subpopulation in RCC tumor tissues, suggesting its potential role in RCC malignant progression and its possible use as a prognostic biomarker for RCC patient prognosis and personalized treatment.

### Overview of cell-cell communication networks

**Figure 8.**
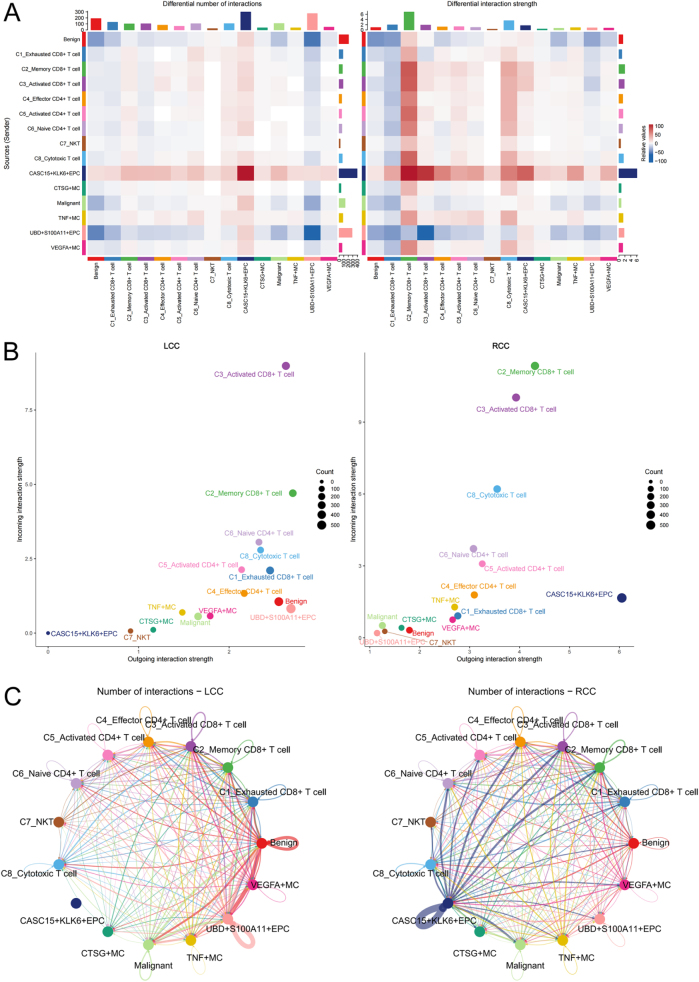
Overview of cell-cell communication networks. Communication intensity between benign and malignant epithelial cells, T cells, and mast cell subpopulations in the integrated data (CellChat analysis; intensity = sum of ligand-receptor probabilities). Differences in outgoing/incoming signal strength. Differential pathway numbers between cell subpopulations (line width proportional to pathway count).

To further elucidate the immune escape mechanisms in the TME, we next explored the intercellular communication network. Using CellChat, we inferred potential cell-cell communication networks based on ligand-receptor co-expression. Although this approach does not directly prove functional interactions, it provides a systematic framework to identify candidate signaling pathways for further validation. In RCC, we observed an increased proportion of exhausted CD8^+^ T cell subsets, along with a decreased proportion of TNF^+^ MCs, suggesting the presence of a suppressive immune microenvironment within RCC tumor tissues. Intercellular interactions, particularly communication between epithelial cells and immune cells, likely play a critical role in tumor immune evasion and progression. Therefore, in-depth analysis of the communication mechanisms between different cell subsets will help reveal how immune cells interact with EPCs in the TME and influence the immune response. To this end, we utilized the R package CellChat (version 1.6.1) to infer the communication relationships between cells. By summarizing the communication probability of all ligand-receptor interactions associated with each signaling pathway, we calculated the communication intensity at the pathway level. Initially, we evaluated the number and strength of intercellular communication pathways between benign and malignant epithelial cells, T cells, and MC subpopulations within the integrated data (Fig. 8A). The results showed that CASC15^+^KLK6^+^EPC exhibited the most active communication with other cell subpopulations, both in terms of the number and intensity of communication. Although Memory CD8^+^ T cells demonstrated more significant communication intensity with other cell subpopulations, the interaction intensity between CASC15^+^KLK6^+^EPC and Memory CD8^+^ T cells was particularly striking, suggesting that CASC15^+^KLK6^+^EPC may play a pivotal role in the TME. Through strong intercellular communication, this subpopulation could potentially regulate the memory and response characteristics of memory CD8^+^ T cells, thereby influencing the immune escape mechanisms of the tumor. Further comparison of the communication signal numbers and strengths between the aforementioned cell subpopulations in LCC and RCC revealed that RCC exhibited significantly higher overall communication intensity than LCC. In RCC, CASC15^+^KLK6^+^EPC not only showed the most significant signal sending intensity but also the highest communication quantity. This specific localization further supports its potential role in RCC. In contrast, UBD^+^S100A11^+^EPC exhibited the highest communication quantity in LCC, with communication intensity second only to memory CD8^+^ T cells and activated CD8^+^ T cells. These results suggest that in the RCC microenvironment, CASC15^+^KLK6^+^EPC might enhance its malignant features and adaptability through a robust intercellular communication network, promoting tumor growth and metastasis. Additionally, memory CD8^+^ T cells and activated CD8^+^ T cells, as the cell populations receiving the most significant signal intensity, may play a critical role in immune response within RCC.

### Differences in communication pathways between LCC and RCC

**Figure 9.**
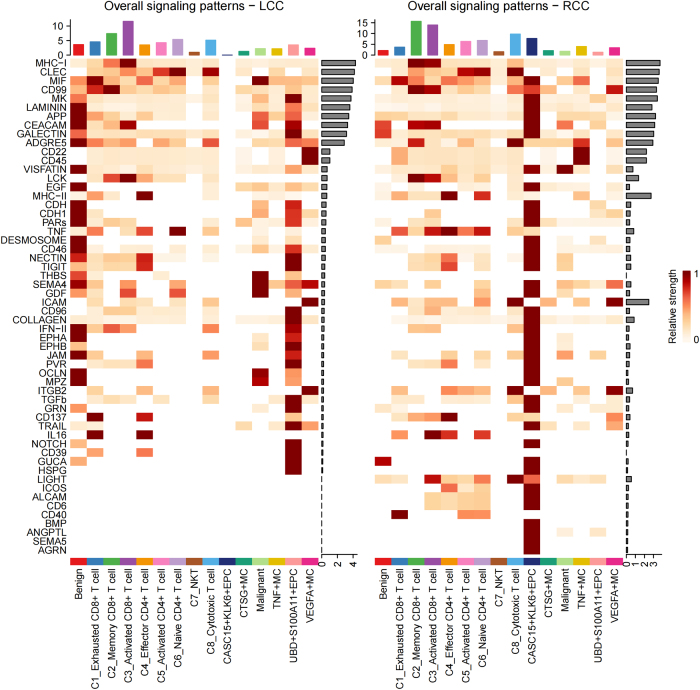
Differences in communication pathways between LCC and RCC.

We further inferred the differences in the intensity of molecular signaling pathways between cell subpopulations in LCC and RCC. The heatmap in Figure 9 displays the communication intensity of 56 major signaling molecules between cell subpopulations in LCC and RCC. The results showed that in LCC, UBD^+^S100A11^+^EPC exhibited significantly higher communication intensity than other cell subpopulations. In contrast, in RCC, CASC15^+^KLK6^+^EPC displayed significantly enhanced communication intensity in most signaling pathways. Particularly in RCC, CASC15^+^KLK6^+^EPC showed significantly increased communication intensity in the LIGHT, ICOS, ALCAM, CD6, CD40, BMP, ANGPTL, SEMA5, and AGRN pathways. LIGHT (Lymphotoxin-Like Inducible Protein 30 kDa), a member of the TNF superfamily, and ICOS (Inducible T-Cell Co-Stimulator), a T-cell co-stimulatory molecule, both show enhanced signaling, which may indicate a robust inflammatory response and immune cell activation within the RCC TME, which not only promotes the aggregation of immune cells but also helps tumor cells evade immune surveillance. The activation of ALCAM (Activated Leukocyte Cell Adhesion Molecule) and CD6 signaling may suggest the enhancement of tumor cell adhesion and migration, which may help tumor cells infiltrate and metastasize to other tissues. In addition, upregulation of BMP (Bone Morphogenetic Protein), ANGPTL (Angiopoietin-Like Protein), and SEMA5 (Semaphorin 5) signals can promote angiogenesis and microenvironment adaptation, ensuring that tumor cells receive adequate nutritional support to enhance their metastatic potential. The enhancement of Agrin signal can further change the matrix structure of TME and provide support for tumor cell attachment and invasion. Collectively, these enhanced communication signals may grant RCC cells a significant growth and invasive advantage within the microenvironment. Notably, the involvement of the CASC15^+^KLK6^+^ EPC subset likely contributes to the development of the malignant phenotype and the increased metastatic potential, which may be one of the reasons behind the poor prognosis in RCC patients.

Heatmap of differentially activated signaling pathways of cell subpopulations in LCC and RCC. Red boxes highlight pathways significantly enhanced.

### EPC and T cell interactions

**Figure 10.**
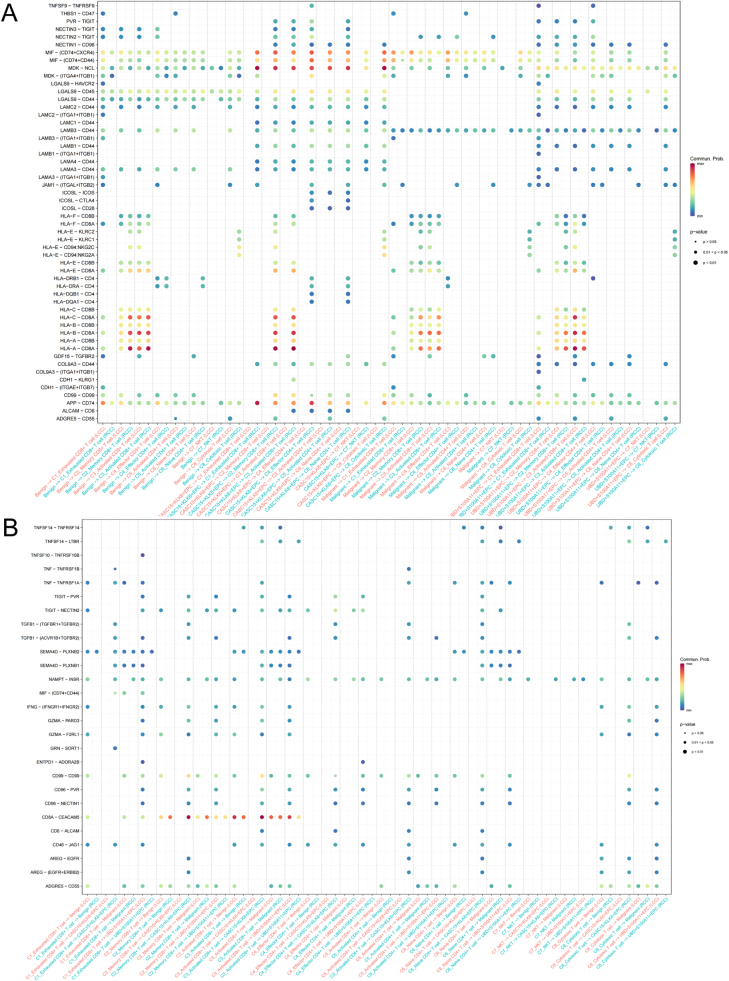
EPC and T cell interactions. (A) Communication from EPC to T cell subpopulations. (B) Communication from T cell to EPC subpopulations. Note: All communications are inferences based on ligand-receptor co-expression.

In our previous analysis, we inferred the differences in the signaling pathways of cell-to-cell communication between various subpopulations in the LCC and RCC microenvironments. Notably, the differential communication strength between EPCs, T cells, and MCs reflects the immune regulatory characteristics of the TME. Next, we further explored the specific communication mechanisms between EPCs and T cells, uncovering the differences in the interactions between different T cell subpopulations and EPC subpopulations in LCC and RCC. The results (Fig. 10A and B) indicated that CASC15^+^KLK6^+^EPC mainly communicates with exhausted CD8^+^ T cells and effector CD4^+^ T cells through the APP-CD74 pathway. It also interacts with memory CD8^+^ T cells and activated CD8^+^ T cells via the HLA-A/B/C-CD8A pathway, as well as through MIF-(CD74^+^CXCR4), MDK-NCL pathways with various T cell subpopulations. Furthermore, in RCC, the communication intensity between benign and UBD^+^S100A11^+^EPC and memory CD8^+^ T cells and activated CD8^+^ T cells through the HLA-A/B/C-CD8A pathway was significantly enhanced compared to LCC (Fig. 10A). In T cell subpopulation communication with EPC subpopulations, memory CD8^+^ T cells mainly communicate with various EPC subpopulations through the CD8A-CEACAM5 (Carcinoembryonic Antigen-Related Cell Adhesion Molecule 5) pathway, with stronger communication in RCC, which may be related to the higher proportion of memory CD8^+^ T cells in RCC. Meanwhile, in LCC, activated CD8^+^ T cells communicated more intensely with various EPC subpopulations through the CD8A-CEACAM5 pathway (Fig. 10B). These results reveal the complex interactions between different types of T cells and EPCs, suggesting that the interactions between EPCs and T cells in the RCC microenvironment are more active, potentially facilitating immune escape and tumor progression. This provides important insights into the malignant characteristics of RCC and potential therapeutic targets.

### T cell and MC communication

**Figure 11.**
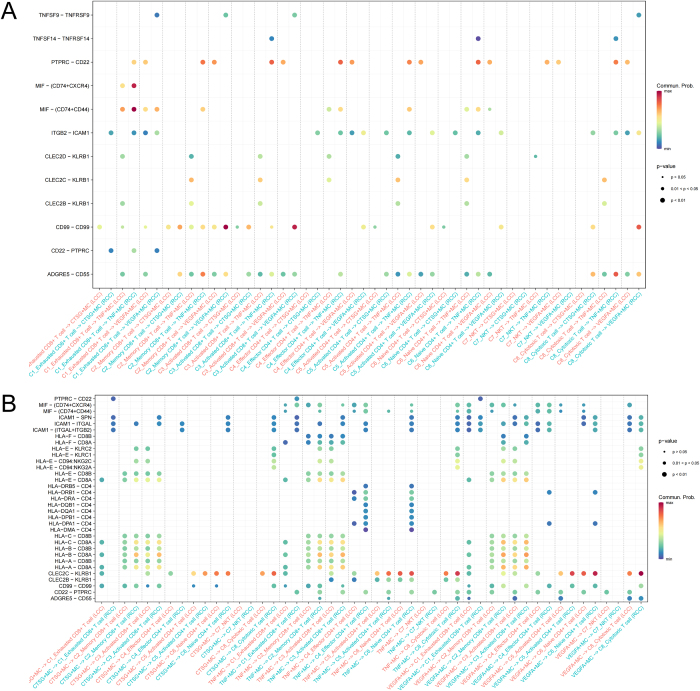
T cell and mast cell communication. (A) Communication from T cell to mast cell subpopulations. (B) Communication from mast cell to T cell subpopulations.

Besides, in the communication analysis between T cell subpopulations and MC subpopulations (Fig. 11A), we observed that MIF and CD99, key signaling molecules closely associated with tumor immune evasion, are involved in the active communication between T cell and MC subpopulations. Specifically, in RCC, exhausted CD8^+^ T cells communicated more strongly with TNF^+^MCs through MIF-(CD74^+^CXCR4) and MIF-(CD74^+^CD44) pathways, while memory CD8^+^ T cells and activated CD8^+^ T cells showed significantly enhanced communication with VEGFA^+^MCs through the CD99-CD99 pathway. As key signaling molecules involved in tumor immune escape, immune suppression, tumor cell migration, and angiogenesis, the enhanced MIF and CD99 signaling pathways in RCC indicate that the interaction between immune cells and MCs in the TME may significantly promote immune escape and angiogenesis, potentially contributing to poor prognosis in RCC patients. MC communication with T cells was most active through the CLEC2C-KLRB1 signaling pathway, especially with activated CD4^+^ T cells and cytotoxic T cells, potentially promoting their activation and cytotoxicity, with more pronounced effects in RCC (Fig. 11B). Additionally, compared to other subpopulations, TNF^+^MCs also communicated with activated CD4^+^ T cells and cytotoxic T cells through multiple HLA-D molecules, facilitating antigen presentation. These pathway communications were more prominent in RCC. However, earlier analysis showed a decrease in the proportion of TNF^+^MCs in RCC, while the proportion of activated CD4^+^ T cells significantly increased and the proportion of effector CD4^+^ T cells decreased. These results suggest that although some immune suppressive factors in RCC reduce the proportion of TNF^+^MCs, their immune regulatory function may not be diminished and may even support the increase of activated CD4^+^ T cells. However, this activation may not translate into the formation of potent effector CD4^+^ T cells due to the dominant immune suppressive factors in the RCC microenvironment. These findings suggest that the RCC microenvironment has complex immune regulatory features, with an increase in activated CD4^+^ T cells, a decrease in TNF^+^MCs, and a reduction in effector CD4^+^ T cells, indicating enhanced immune suppression, and potentially providing tumor cells with an advantage in immune evasion.

### EPC and MC interactions

**Figure 12.**
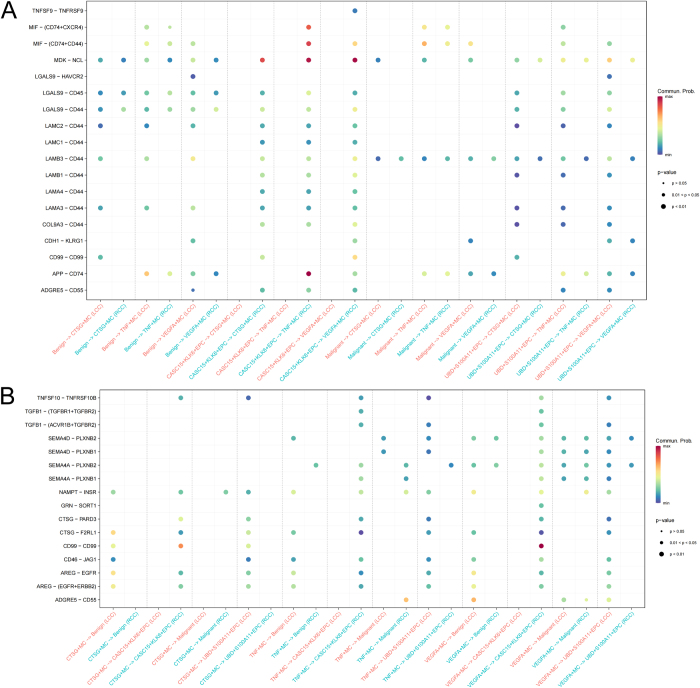
EPC and mast cell interactions. (A) Communication from EPC to mast cell subpopulations. (B) Communication from mast cell to EPC subpopulations.

The interaction between EPC and MC was further analyzed (Fig. 12A), we found that CASC15^+^KLK6^+^EPC communicated strongly with TNF^+^MCs via APP-CD74, MIF-(CD74^+^CXCR4), and MIF-(CD74^+^CD44), which may be associated with immune escape in the RCC microenvironment. CASC15^+^KLK6^+^EPC also communicated with VEGFA^+^MCs through the MIF-(CD74^+^CXCR4) and APP-CD74 pathways. Further analysis of this communication demonstrated that in RCC, communication intensity between various EPC subpopulations and MC subpopulations significantly increased, especially in the communication between CASC15^+^KLK6^+^EPC and TNF^+^MCs, VEGFA^+^MCs, and CD99^+^MCs, compared to LCC. This could suggest that EPC subpopulations, especially CASC15^+^KLK6^+^EPC, might play a crucial role in the promotion of immune escape and metastasis in RCC through these robust communication pathways.

Moreover, based on the above analysis of the pairwise communication between EPCs, T cells, and MCs, we also discovered that MIF (Macrophage Migration Inhibitory Factor) and CD99 act as key signaling molecules significantly involved in the interaction network between EPCs, T cells, and MCs. MIF likely regulates the communication between EPCs, T cells, and MCs through its binding with receptors such as CD74, CXCR4, and CD44, thereby mediating immune evasion[29]. Similarly, the CD99 pathway, which is closely linked to immune evasion, angiogenesis, and invasion in the TME[30], prominently connects the interactions among EPCs, T cells, and MCs. The combined action of these signaling molecules may enhance tumor cell immune escape and growth advantage, thereby driving the malignant progression of RCC and contributing to poor patient prognosis. Therefore, targeting the MIF and CD99 signaling pathways may offer novel therapeutic strategies and potential targets for RCC treatment.

## Discussion

Previous research has demonstrated significant differences in epidemiology, clinical features, tumor identification, treatment response, prognosis, and molecular characteristics betweenRCC and LCC^[5-8,31–33]^. CRC sidedness has emerged as a critical determinant of prognosis, treatment response, and molecular characteristics. While previous studies using bulk transcriptomics have established distinct features between RCC and LCC tumors, these approaches lacked the resolution to identify functional subpopulations and infer specific cell–cell interactions. In this study, we applied single-cell RNA sequencing to dissect the immune microenvironment and epithelial heterogeneity of LCC and RCC, with a focus on T cells, MCs, and malignant epithelial cells.

T cells were the predominant immune population in both LCC and RCC, in line with prior findings^[14,15]^. Our study revealed a higher abundance of exhausted CD8^+^ T cells in RCC. While previous studies have linked CD8^+^ T cell exhaustion with tumor immune evasion[34], this study may reinforce RCC’s immunosuppressive nature and its association with ineffective anti-tumor immunity. In contrast, LCC exhibited relatively lower immune exhaustion, suggesting that its immune microenvironment may be more responsive. This difference may be related to variations in molecular mechanisms, microenvironmental factors, and genetic backgrounds.

MCs have emerged as key regulators of angiogenesis and immune suppression[23]. but their role in RCC-specific microenvironment remodeling was understudied. MCs play multiple roles in the tumor immune microenvironment, enhancing immune cell aggregation through the secretion of pro-inflammatory factors such as TNF (TNF^+^MC) and promoting tumor growth through the secretion of angiogenic factors (VEGFA^+^MC).^[18–21]^ In our dataset, RCC was characterized by a decrease in TNF^+^ MCs and an increase in VEGFA^+^ MCs, suggestive of a shift toward pro-angiogenic and immunosuppressive functions. This shift aligns with recent studies indicating that MC subtypes play differential roles in tumor progression[21]. Our data provide the first single-cell evidence of MC polarization in CRC sidedness and implicate their potential role in RCC progression.

Epithelial cell malignant transformation is a key step in the development of CRC[35]. Our study identified a CASC15^+^KLK6^+^ epithelial subpopulation enriched in RCC. These cells exhibited copy number variation (CNV) and high expression of genes associated with proliferation, invasion, and metastasis.CASC15 has been shown to be abnormally highly expressed in various tumors and can affect tumor proliferation, invasion, and apoptosis.^[36–38]^ Kallikrein-related peptidase 6 (KLK6) is a secreted serine protease that plays an important role in tumor growth, invasion, and metastasis.^[39–41]^ Previous studies have reported that the expression of KLK6 mRNA in colon cancer (CC) is significantly correlated with increased tumor stage and histological grade[42]. But our analysis suggests that their co-expression defines a highly malignant epithelial lineage. While a recent single-cell sequencing analysis study has found that MM7 + cancer cells promoted left-sided CRCs metastasis[43]. Our pseudotime analysis indicated that these cells likely evolve from homeostatic enterocytes, with downregulation of GUCA2B and TSPAN1 contributing to loss of epithelial identity. Previous studies have indicated that the dysregulation of GUCA2A is a promising biomarker for accurate diagnosis and prognosis of CRC[44], and TSPAN1, a new member of the tetraspanin family, has also been shown to be highly involved in carcinogenesis and chemoresistance[45]. These findings offer a new perspective for understanding the mechanisms underlying CRC development. Considering our limited sample size (n =3 per group), we verified the main findings in a larger cohort (n = 30/40 per group), and more, a negative correlation (r = − 0.2919, p = 0.0142) between CASC15^+^KLK6^+^ cell density and patient survival supports their potential as prognostic markers.

Using CellChat, we inferred that CASC15^+^KLK6^+^ epithelial cells interact extensively with exhausted and memory CD8^+^ T cells as well as VEGFA^+^ MCs. RCC displayed enhanced signaling via MIF-(CD74^+^CXCR4) and CD99-CD99 ligand-receptor pairs. These pathways are previously implicated in immune escape and angiogenesis^[29,30]^ but not described in sidedness-specific contexts. Although our findings are based on transcriptomic inference, they highlight novel epithelial-immune-stromal interactions that may drive RCC progression.

The findings of this study highlight the significant differences between LCC and RCC in their immune microenvironments. RCC exhibits stronger immunosuppressive and immune evasion characteristics, which may be closely related to its poor prognosis. The distinct immune microenvironment of RCC may stem from multiple factors. Embryologically, the right and left colon derive from different gut segments (midgut vs hindgut), which could imprint differential stromal-epithelial crosstalk. Additionally, the right colon harbors a denser microbiome with distinct composition, potentially driving TLR-mediated inflammation[17]. Future studies should investigate how microbial metabolites interact with KLK6^+^ epithelial cells to promote immunosuppression.

Our work presents a single-cell landscape that quantifies T cell exhaustion, MC polarization, and epithelial diversification in the context of tumor laterality. These findings not only support earlier observations but also identify CASC15^+^KLK6^+^ cells and MIF/CD99 signaling as novel features potentially amenable to therapeutic intervention. However, the small sample size in our scRNA-seq cohort is a significant limitation, as CRC is known for its intertumoral heterogeneity. Although we validated the key finding of CASC15^+^KLK6^+^EPC expansion in RCC using a larger cohort, future studies with larger sample sizes are needed to confirm the robustness of other observations, such as the differences in T cell exhaustion and MC reprogramming. Future research could further explore the potential of these cellular subpopulations as clinical biomarkers and the possibility of improving immune therapy responses in RCC patients by targeting intercellular communication pathways.

## Conclusion

While our study provides novel insights into the immune microenvironment of RCC versus LCC, the small sample size of the scRNA-seq discovery cohort necessitates cautious interpretation. We identified RCC-specific features including enhanced T cell exhaustion, pro-angiogenic and immunosuppressive MC polarization, and a malignant CASC15^+^KLK6^+^ epithelial subpopulation that may drive tumor progression through MIF and CD99-mediated intercellular communication. These findings generate new hypotheses about the immunosuppressive landscape of RCC and highlight candidate pathways for therapeutic intervention. Future studies with expanded sample sizes, functional validation, and integration of additional data types (e.g., microbiome, spatial transcriptomics, lineage tracing) will be critical for confirming and expanding upon these findings.

## Data Availability

The sequencing data generated in this study have been deposited in the Sequence Read Archive (SRA) under accession number SRP557106 and are available at NCBI BioProject: PRJNA1210001.

## Research Registration Unique Identifying Number (UIN)

This study has been registered and filed with the China Medical Research Registration and Record Filing Information System (Registration Number: MR-33-23-020761).
